# Supplementary material for: Reduced marine phytoplankton sulphur emissions in the Southern Ocean during the past seven glacials
Source: Nat Commun. 2019 Jul 19;10:3247. doi: 10.1038/s41467-019-11128-6 (PMC6642193; doi:10.1038/s41467-019-11128-6)
Supplement: Supplementary file 1 — Supplemenatary Information [file 41467_2019_11128_MOESM1_ESM.pdf]

Supplementary Information

Reduced marine phytoplankton sulphur emissions in the Southern Ocean during the past seven glacials

K. Goto-Azuma et al.

Supplementary Figures

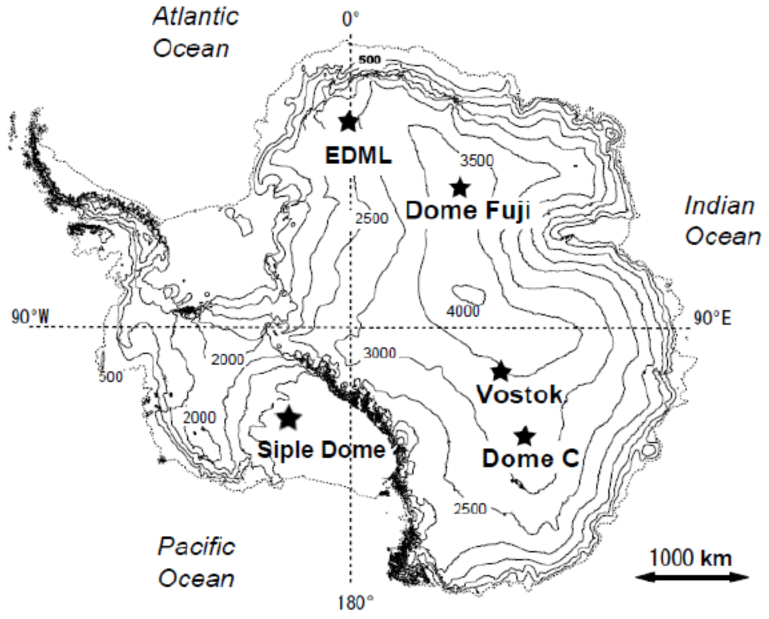

Supplementary Figure 1. Location of Dome Fuji (DF) in East Antarctica. The map is modified after [*1*]. Additional deep ice core sites referred to in the text are marked.

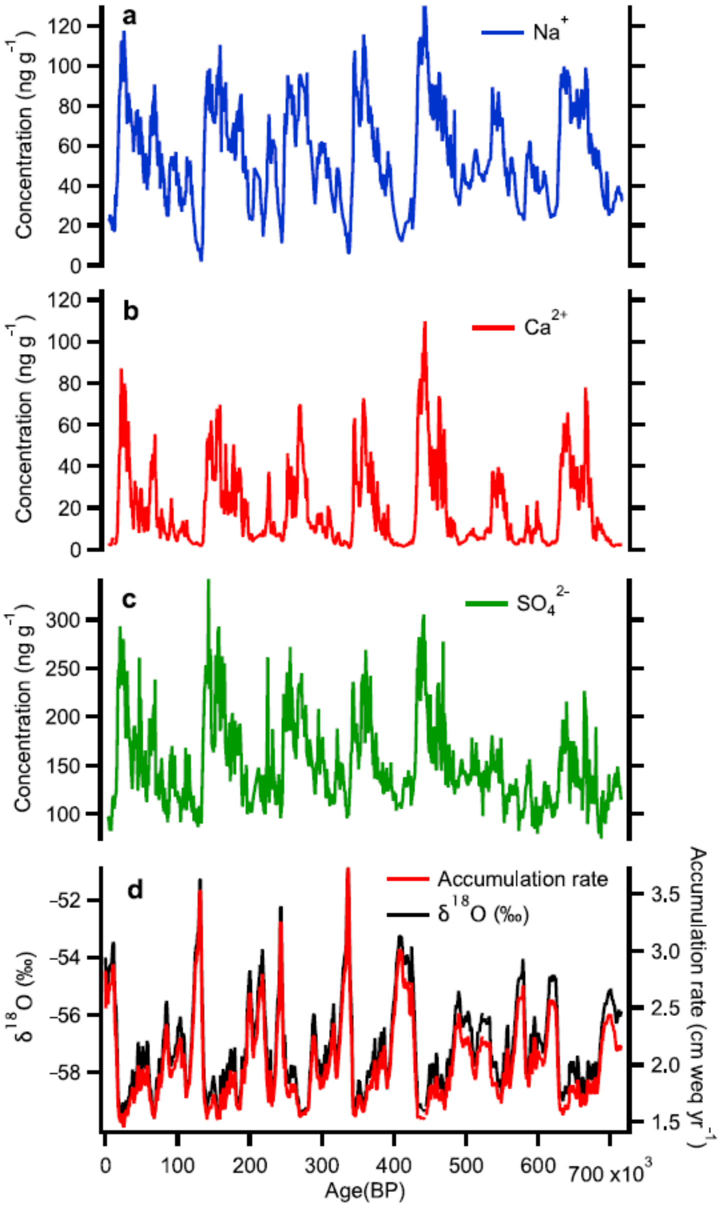

Supplementary Figure 2. Variability in ion concentrations and accumulation rates at Dome Fuji (DF). a Na^+, **b** Ca^{2+}, and **c** SO_4^{2-} concentrations (see Methods). **d** Accumulation rates together with $\delta^{18}O$ at DF[1]. All ion and $\delta^{18}O$ data are averages over 1000 years.

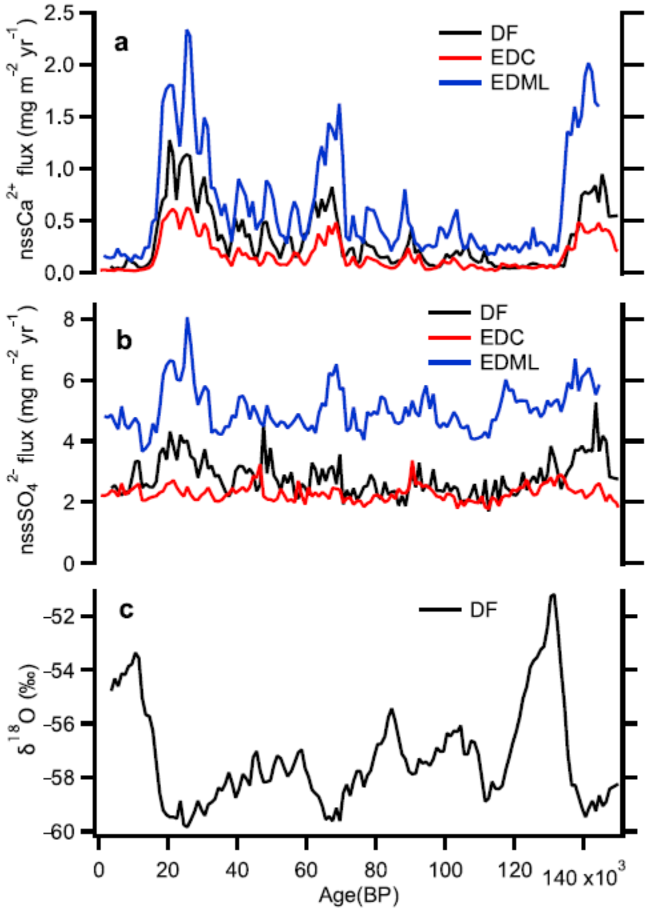

Supplementary Figure 3. Variability in $nssCa^{2+}$ and $nssSO_4^{2-}$ fluxes for the past 150,000 years at Dome Fuji (DF), Dome C (EDC), and Dronning Maud Land (EDML). a $nssCa^{2+}$ at DF, EDC, and EDML, **b** $nssSO_4^{2-}$ at DF, EDC, and EDML, and **c** $\delta^{18}O$ at DF[1]. See Methods for the DF chronology and flux calculations. The EDC and EDML fluxes are plotted on the AICC12 timescale[2, 3] using previously published ion data[4-7] and accumulation rates[2,3]. All ion and $\delta^{18}O$ data are averages over 1000 years.

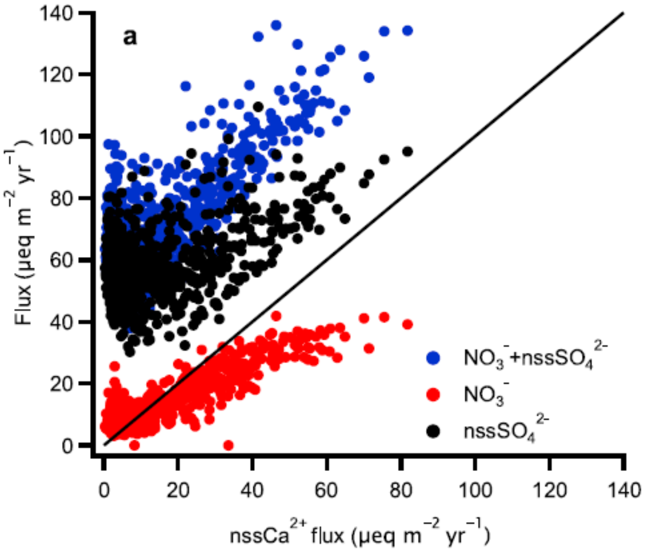

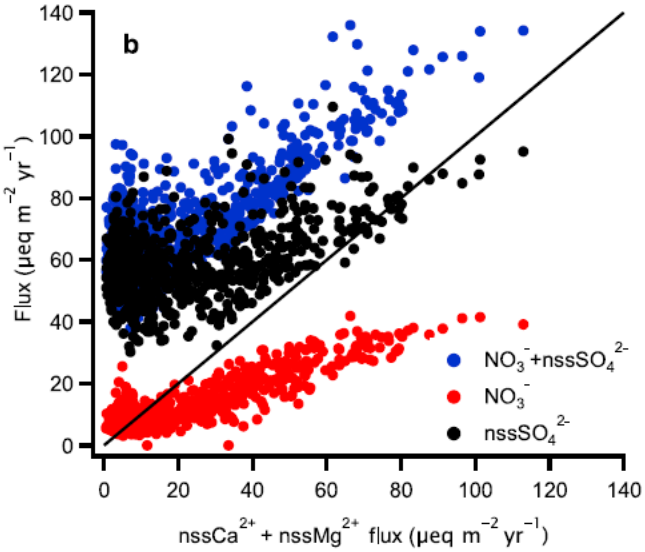

Supplementary Figure 4. Correlation between ion fluxes at Dome Fuji (DF). a $nssSO_4^{2-}$ flux (black), NO_3^- flux (red), and [NO_3^- + $nssSO_4^{2-}$] flux (blue) plotted against $nssCa^{2+}$ flux. **b** $nssSO_4^{2-}$ flux (black), NO_3^- flux (red), and [NO_3^- + $nssSO_4^{2-}$] flux (blue) plotted against [$nssCa^{2+}$ + $nssMg^{2+}$] flux. Black lines represent a stoichiometric ratio of 1. All data are averages over 1000 years. The strong correlation between [NO_3^- + $nssSO_4^{2-}$] flux and [$nssCa^{2+}$ + $nssMg^{2+}$] flux during cold periods supports the assumption that the majority of terrestrial $CaCO_3$, $MgCO_3$, and $CaMg(CO_3)_2$ are converted to $Ca(NO_3)_2$ and $Mg(NO_3)_2$ by HNO_3 or NO_x (see Supplementary Discussion).

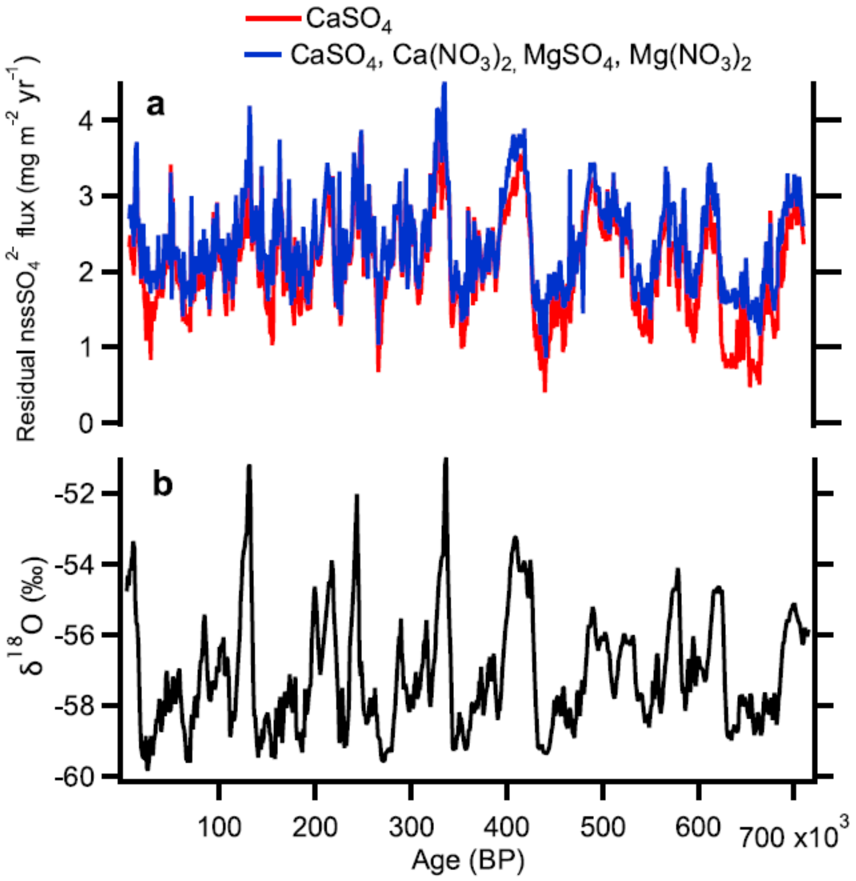

Supplementary Figure 5. Different estimates of marine biogenic $nssSO_4^{2-}$ flux (residual $nssSO_4^{2-}$ flux) at Dome Fuji (DF). a Residual $nssSO_4^{2-}$ flux considering (i) only terrestrial $CaSO_4$ contributions to $nssSO_4^{2-}$ flux (red), and (ii) terrestrial $CaSO_4$ and $MgSO_4$ contributions along with $Ca(NO_3)_2$ and $Mg(NO_3)_2$ (blue). **b** $\delta^{18}O$ at DF[1]. All ion and $\delta^{18}O$ data are averages over 1000 years.

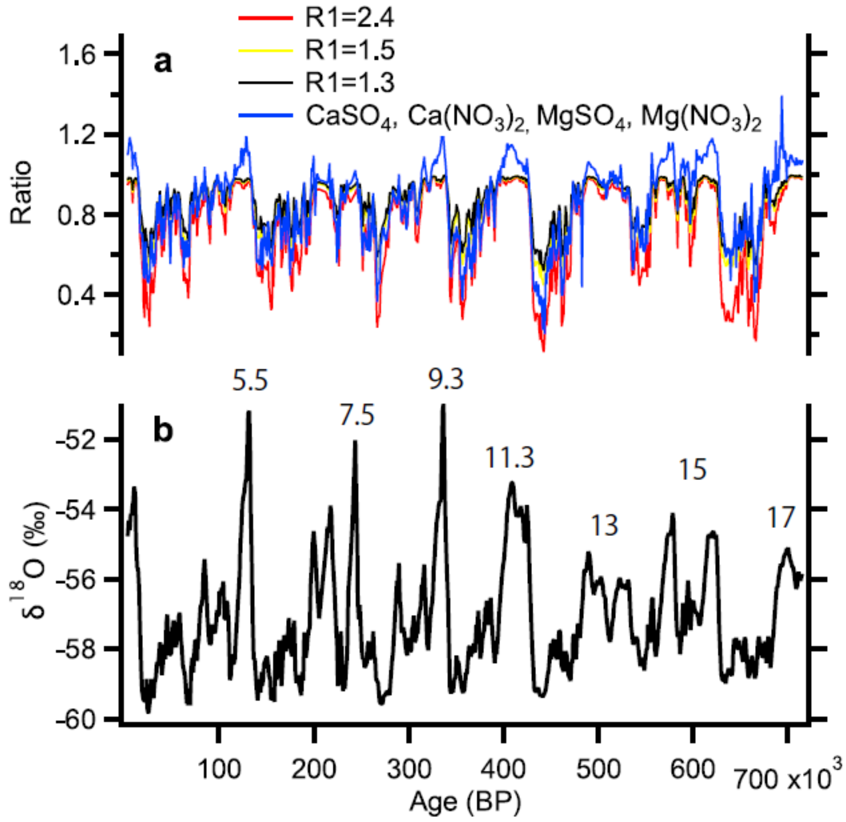

Supplementary Figure 6. Marine biogenic/total $nssSO_4^{2-}$ ratios at Dome Fuji (DF) for different estimations of marine biogenic sulphate. a Ratios (R_2) calculated based on different estimations. Marine biogenic sulphate was estimated considering (i) only terrestrial $CaSO_4$ contributions to the $nssSO_4^{2-}$ flux with terrestrial $nssSO_4^{2-}/nssCa^{2+}$ ratio (R_1) = 2.4 (red), (ii) same as (i) but with R_1 = 1.5 (yellow), (iii) same as (i) but with R_1 = 1.3 (black), and (iv) terrestrial $CaSO_4$ and $MgSO_4$ contributions along with $Ca(NO_3)_2$ and $Mg(NO_3)_2$ (blue). **b** $\delta^{18}O$[1]. Ratios and $\delta^{18}O$ data are averages over 1000 years. Marine isotope stage numbers for interglacials are also shown.

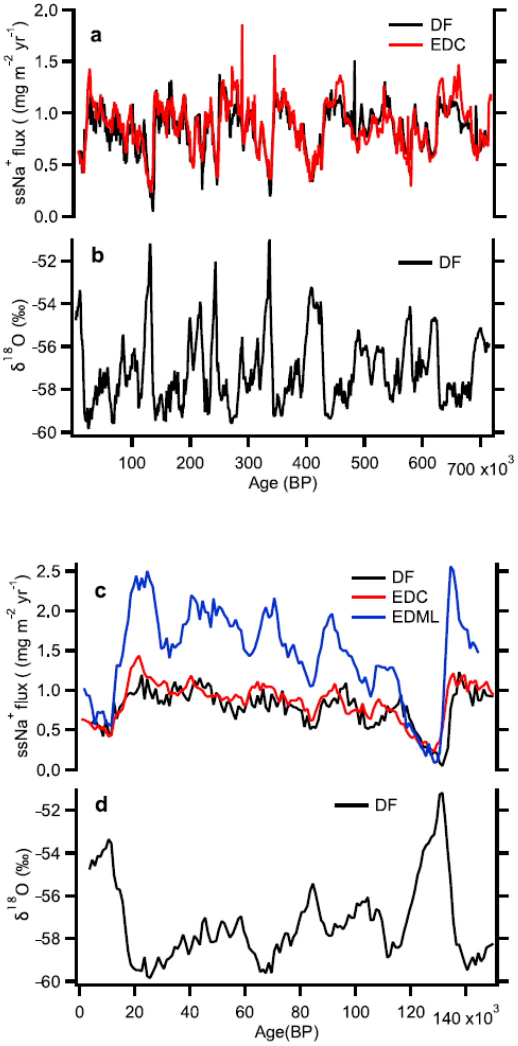

Supplementary Figure 7. Comparison of $ssNa^+$ fluxes at Dome Fuji (DF), Dome C (EDC), and Dronning Maud Land (EDML). a Flux of $ssNa^+$ at DF and EDC over the past 720,000 years. **b** $\delta^{18}O$[1] at DF over the past 720,000 years. **c** Flux of $nssNa^+$ at DF, EDC, and EDML over the past 150,000 years. **d** $\delta^{18}O$[1] at DF over the past 150,000 years. The EDC and EDML fluxes are plotted on the AICC12 timescale[2,3] using previously published ion data[4,5,7] and accumulation rates[2,3]. All data are averages over 1000 years. The similarity between $ssNa^+$ flux at DF and EDC suggests that both sites are affected by inland air masses, which are likely a mixture of air masses from different oceanic sectors[8,9].

Supplementary Discussion

Different estimations of residual $nssSO_4^{2-}$

We examine the contributions of terrestrial cations other than Ca^{2+}. $NssK^+$ and $nssMg^{2+}$ could originate from sulfate-containing mineral dust. We calculate equivalent concentrations of $nssK^+$ and $nssMg^{2+}$ where $[nssK^+] = [K^+] - 0.02\cdot [ssNa^+]$, and $[nssMg^{2+}] = [Mg^{2+}] - 0.11\cdot [ssNa^+]$, using equivalent seawater ratios of $K^+/Na^+ = 0.02$ and $Mg^{2+}/Na^+ = 0.11$. We use [ion] for the equivalent concentration/flux of each ion. Much lower equivalent concentrations of $nssK^+$ compared with $nssCa^{2+}$ (less than 5% for most of DF2 core, not measured for DF1 core) suggest that contributions of K-containing minerals are much lower than those of $CaSO_4$. Equivalent concentrations of $nssMg^{2+}$ are correlated with those of $nssCa^{2+}$, with the former being about 35% of the latter. This suggests that during cold periods in glacials, a major part of $nssMg^{2+}$ could also originate from evaporites in the form of $MgSO_4$ or $CaMg(CO_3)_2$, as deduced from the strong correlation between the Mg^{2+} and SO_4^{2-} fluxes. To remove terrestrial $MgSO_4$ contributions from the $nssSO_4^{2+}$ flux, we should subtract the $nssMg^{2+}$ only associated with $nssSO_4^{2-}$.

It should be noted that South American dust source areas include $CaCO_3$-rich sources[10], as well as gypsum-rich sources[11,12]. Because $CaCO_3$ is not a major chemical form of Ca^{2+} in ice cores from Antarctic interior sites[13], previous studies assume that $CaCO_3$ reacts with H_2SO_4 or SO_2 and forms $CaSO_4$[14,15]. Ca^{2+}, however, has also been reported to be associated with NO_3^- in the Antarctic interior during cold periods in glacials when dust concentrations increases[16-18]. Supplementary Fig. 4a displays a strong correlation between the NO_3^- flux and $nssCa^{2+}$ flux at DF, which indicates the presence of $Ca(NO_3)_2$ at DF. Recent studies show that $CaCO_3$ reacts more readily with HNO_3 or NO_x than with SO_2 or H_2SO_4 and that the reaction with SO_2 or H_2SO_4 is very slow[16,19-22]. We therefore deduce that primary continental gypsum is a major

contributor to the $CaSO_4$ flux and that secondary $CaSO_4$, formed by the reaction between $CaCO_3$ and H_2SO_4/SO_2, is only a minor contributor. Here, we assume that $CaCO_3$ completely reacts with HNO_3/NO_x and that $CaSO_4$ originates solely from continental gypsum. Similarly, some $nssMg^{2+}$ would be associated with NO_3^{-} because $MgCO_3$ and $CaMg(CO_3)_2$, as well as $CaCO_3$, react readily with HNO_3/NO_x and form $Mg(NO_3)_2$[19]. A strong correlation between $nssMg^{2+}$ flux and NO_3^{-} flux at DF provides evidence of this reaction. Using the following equation, we can better estimate residual $nssSO_4^{2-}$ concentrations $[nssSO_4^{2-}]_{res}$, a revised proxy for marine biogenic sulphate, because $nssCa^{2+}$ and $nssMg^{2+}$ are both associated with NO_3^{-}.

$$[nssSO_4^{2-}]_{res} = [nssSO_4^{2-}] - ([nssCa^{2+}] + [nssMg^{2+}] - [NO_3^{-}])$$

Here we assume that the majority of terrestrial $CaCO_3$, $MgCO_3$, and $CaMg(CO_3)_2$ are converted to $Ca(NO_3)_2$ and $Mg(NO_3)_2$ by HNO_3/NO_x. This assumption is supported by a strong correlation between the $[NO_3 + nssSO_4^{2-}]$ flux and $[nssCa^{2+} + nssMg^{2+}]$ flux at DF during cold periods with increased dust flux and by the slope (~1) of the lower bound of the scatter plot (Supplementary Fig. 4b).

Although residual $nssSO_4^{2-}$ fluxes calculated with different assumptions give different r^2 values (Fig. 4), both decrease during cold periods in glacials. Interestingly, the first-order approximation, assuming that $nssCa^{2+}$ is associated only with $nssSO_4^{2-}$ and that $nssSO_4^{2-}$ is associated only with $nssCa^{2+}$ during cold periods in glacials, is similar to the estimation of residual $nssSO_4^{2-}$ flux using the equation above. Although $nssMg^{2+}$ and NO_3^{-} data are not available for EDC and EDML cores, it is expected that the calculation of residual $nssSO_4^{2-}$ using only $nssCa^{2+}$ and $nssSO_4^{2-}$ would be fairly reasonable.

Causes of reduced biogenic sulphate flux during glacials

A tracer transport model using the DMS concentration data of [*23*] shows that in austral summer, more than 60% (80%) of the geographic origin of DMS leading to sulphate at Vostok (Supplementary Fig. 1), an East Antarctica interior site, is located south of 55°S (50°S)[24]. Although the climatology of summer DMS concentrations has been recently revised, mean summer DMS concentrations in the SO are close to the original estimates[23,25]. The Antarctic Polar Front, the northern edge of the AZ, lies roughly between 50°S and 60°S[26]. These studies suggest that a major source region of DMS for the Antarctic interior is the AZ. A study of sulphur cycling at the LGM based on atmospheric general circulation and sulphur chemistry models indicates that biogenic sulphate flux on the Antarctic ice sheet is sensitive to both oceanic DMS concentration and spatial distribution[27]. The latter strongly depends on the extent of summer sea ice[27]. The model study also shows that the flux is almost proportional to oceanic DMS concentrations, provided that the summer sea ice extent does not change. The proportion of DMS oxidized to SO_4^{2-} or MSA can vary with time, but SO_4^{2-} likely remains the dominant product[4,27]. Changes in atmospheric circulation and DMS oxidation chemistry have minor impacts on the model results. Depositional processes do not change substantially under different climate conditions because dry deposition remains the dominant process at the Antarctic interior sites discussed here.

The lid effect of sea ice could move the source region north during glacials as a result of sea ice expansion[27], which would lead to a larger transport distance and lower flux of DMS-derived sulphate. Sea ice reconstructions based on ocean sediment cores suggest that while the winter LGM sea ice extent is about two times greater than the present day, the LGM summer extent appears to be only slightly greater, except for the Weddell Sea area, where the occurrence of

sporadic sea ice is observed around the present-day winter sea ice edge[28]. DMS-derived sulphur species in EDC, EDML, and Siple Dome ice cores have been traditionally assumed to be dominated by those from the nearby Indian, Atlantic, and Pacific Ocean sectors of the SO, respectively[4-6,29]. Accordingly, DF should be mainly affected by the Atlantic and Indian Ocean sectors. The large difference in summer sea ice extent between the Atlantic and Indian Ocean sectors would lead to differences in the marine biogenic sulphate flux between DF and EDC. However, this is not the case. DF and EDC show rather similar fluxes and variations in the marine biogenic sulphate flux (Fig. 3a).

According to recent back-trajectory studies, the Antarctic interior sites (e.g. DF and EDC) are affected by inland air masses, which are likely a mixture of air masses from different oceanic sectors, as well as by air masses from the ocean sectors they face[8,9]. Thus, the marine biogenic sulphate fluxes at DF and EDC reflect variations in the overall DMS emissions in the AZ. This result is supported by the similarity between $ssNa^+$ fluxes at DF and EDC, which likely originates from the sea ice surface[4,5,7,29] (Supplementary Fig. 7). Overall, the summer sea ice field around Antarctica at the LGM is estimated to be about 1.25 to 1.50 times that of today, although data remain sparse[28]. This finding points to similar or only slightly more distant source areas for DMS during glacials compared to interglacials. Hence, the large glacial/interglacial variability in the marine biogenic sulphate flux at DF and EDC would be dominated by glacial/interglacial variability in DMS emissions in the AZ, rather than changes in transport distance.

EDML, a site close to the Weddell Sea where summer sea ice during glacials seems to have expanded much farther compared to other ocean sectors[28], shows higher biogenic sulphate fluxes than DF and EDC (Fig. 3c), as well as higher $ssNa^+$ fluxes (Supplementary Fig. 7c). This can be

explained by more efficient transport of marine air masses to EDML than to the sites located farther inland (DF and EDC)[8,9]. Reduced marine biogenic sulphate flux at EDML during glacials could be a result of the longer transport distance from the summer sea ice edge. Reduced DMS emissions during glacials should also play a role considering the strong DMS emissions from leads and polynyas in the present-day summer sea ice zone[29]. Although we cannot yet conclude which of the two causes is more important, the coherent variability in marine biogenic sulphate fluxes at EDML, DF, EDC, and Siple Dome suggests reduced DMS emissions in the Atlantic Ocean sector, as well as in other ocean sectors.

To evaluate the effect of solar irradiance on DMS emissions, we calculated the integrated summer insolation at 55°S, the latitude of a major source region of DMS[24]. Since the modern daily insolation at the spring and autumnal equinox at 55°S is about 250 W/m^2 [30], we integrated daily insolation >250 W/m^2 over the year[31]. We calculated power spectra with the Blackman-Tukey method (30% lag) using the Analyseries software package[32]. Although strong powers were found in the 41-kyr and 98-kyr bands, similar to those for $\delta^{18}O$, the variability was less than 3%. Integrated summer insolations at 50°S, 60°S, 65°S, and 70°S give similar power spectra as 55°S. Variability of the integrated summer insolation from 50°S to 70°S was 3%–10%, depending on the latitude. This small variability would not be a major cause of the variability in DMS emissions[33].

Supplementary References

1. Dome Fuji Ice Core Project members, State dependence of climatic instability over the past 720,000 years from Antarctic ice cores and climate modeling. *Sci. Advances* **3**, doi: 10.1126/sciadv.1600446 (2017).

2. Bazin, L. et al. An optimized multi-proxy, multi-site Antarctic ice and gas orbital chronology (AICC2012): 120–800 ka. *Clim. Past* **9**, 1715–1731 (2013).

3. Veres, D. et al. The Antarctic ice core chronology (AICC2012): an optimized multi-parameter and multi-site dating approach for the last 120 thousand years. *Clim. Past* **9**, 1733–1748 (2013).

4. Wolff, E. W. et al. Southern Ocean sea-ice extent, productivity and iron flux over the past eight glacial cycles. *Nature* **440**, 491–496 (2006).

5. Wolff, E. W. et al. Changes in environment over the last 800,000 years from chemical analysis of the EPICA Dome C ice core. *Quat. Sci. Rev.* **29**, 285–295 (2010).

6. Kaufmann, P. et al. Ammonium and non-sea salt sulfate in the EPICA ice cores as indicators of biological activity in the Southern Ocean. *Quat. Sci. Rev.* **29**, 313–323 (2010).

7. Fischer, H. et al. Reconstruction of millennial changes in dust emission, transport and regional sea ice coverage using the deep EPICA ice cores from the Atlantic and Indian Ocean sector of Antarctica. *Earth Planet. Sci. Lett.* **260**, 340–354 (2007).

8. Suzuki, K., Yamanouchi, T. & Motoyama, H. Moisture transport to Syowa and Dome Fuji stations in Antarctica. *J. Geophys. Res.* **113**, D24114 (2008).

9. Suzuki, K., Yamanouchi, T., Kawamura, K. & Motoyama, H. The spatial and seasonal distributions of air-transport origins to the Antarctic based on 5-day backward trajectory analysis. *Polar Sci.* **7**, 205–213 (2013).

10. Bockheim, J. G. & Douglass, D. C. Origin and significance of calcium carbonate in soils of southwestern Patagonia. *Geoderma* **136**, 751–762 (2006).

11. Alonso, R. N., Jordan, T. E., Tabbutt, K. T. & Vandervoort, D. S. Giant evaporite belts of

the Neogene central Andes. *Geology* **19**, 401–404 (1991).

12. Drewry, G. E., Ramsay, A. T. S. & Smith, A. G. Climatically controlled sediments, geomagnetic-field, and trade wind belts in Phanerozoic time. *J. Geol.* **82**, 531–553 (1974).

13. Kawamura, K. et al. Atmospheric CO_2 variations over the last three glacial-interglacial climatic cycles deduced from the Dome Fuji deep ice core, Antarctica using a wet extraction technique. *Tellus B Chem. Phys. Meteorol.* **55**, 126–137 (2003).

14. Legrand, M. R., Lorius, C., Barkov, N. I. & Petrov, V. N. Vostok (Antarctica) ice core: Atmospheric chemistry changes over the last climatic cycle (160,000 years). *Atmos. Environ.* **22**, 317–331 (1988).

15. Oyabu, I. et al. Chemical compositions of sulfate and chloride salts over the last termination reconstructed from the Dome Fuji ice core, inland Antarctica. *J. Geophys. Res. Atmos.* **119**, 14045–14058 (2014).

16. De Angelis, M., Traversi R. & Udisti, R. Long-term trends of mono-carboxylic acids in Antarctica: comparison of changes in sources and transport processes at the two EPICA deep drilling sites. *Tellus B Chem. Phys. Meteorol.* **64**, 1–21 (2012)

17. Rothlisberger, R., Hutterli, M. A., Sommer, S., Wolff, E. W. & Mulvaney, R. Factors controlling nitrate in ice cores: evidence from the Dome C deep ice core. *J. Geophys. Res. Atmos.* **105**, 20565–20572 (2000).

18. Legrand, M. & Mayewski, P. Glaciochemistry of polar ice cores: A review. *Rev. Geophys.* **35**, 219–243 (1997).

19. Usher, C. R., Michel, A. E. & Grassian, V. H. Reactions on mineral dust. *Chem. Rev.* **103**, 4883–4940 (2003).

20. Ooki, A. & Uematsu, M. Chemical interactions between mineral dust particles and acid gases during Asian dust events. *J. Geophys. Res. Atmos.* **10**, doi: 10.1029/2004JD004737 (2005).

21. Sullivan, R. C., Guazzotti, S., Sodeman, D. A. & Prather, K. A. Direct observations of the atmospheric processing of Asian mineral dust. *Atmos. Chem. Phys.* **7**, 1213–1236 (2007).

22. Pan, X. et al. Real-time observational evidence of changing Asian dust morphology with the mixing of heavy anthropogenic pollution. *Sci. Rep.* **7**, 335 (2017).

23. Kettle, A. J. et al. global database of sea surface dimethylsulfide (DMS) measurements and a procedure to predict sea surface DMS as a function of latitude, longitude, and month. *Global Biogeochem. Cycles* **13**, 399–444 (1999).

24. Cosme, E. Origin of dimethylsulfide, non-sea-salt sulfate, and methanesulfonic acid in eastern Antarctica. *J. Geophys. Res.* **110**, D03302 (2005).

25. Jarnikova, T. & Tortell, P. D. Towards a revised climatology of summertime dimethylsulfide concentrations and sea-air fluxes in the Southern Ocean. *Environment. Chem.* **13**, 364–378 (2016).

26. Belkin, I. M. & Gordon, A. L. Southern Ocean fronts from the Greenwich meridian to Tasmania. *J. Geophys. Res. Oceans* **101**, 3675–3696 (1996).

27. Castebrunet, H., Genthon, C. & Martinerie, P. Sulfur cycle at Last Glacial Maximum: model results versus Antarctic ice core data. *Geophys. Res. Lett.* **33**, L22711 (2006).

28. Gersonde, R., Crosta, X., Abelmann, A. & Armand, L. Sea-surface temperature and sea ice distribution of the Southern Ocean at the EPILOG Last Glacial Maximum-a circum-Antarctic view based on siliceous microfossil records. *Quat. Sci. Rev.* **24**, 869–896 (2005).

29. Abram, N. J., Wolff, E. W. & Curran, M. A. J. A review of sea ice proxy information from polar ice cores. *Quat. Sci. Rev.* **79**, 168–183 (2013).

30. Laskar, J. et al. A long-term numerical solution for the insolation quantities of the Earth. *Astron. Astrophys.* **428**, 261–285 (2004).

31. Huybers, P. Early Pleistocene glacial cycles and the integrated summer insolation forcing. *Science* **313**, 508–511 (2006).

32. Paillard, D., Labeyrie, L. & Yiou, P. Macintosh program performs time-series analysis. *Eos Trans. Amer. Geophys. Union* **77**, 379 (1996).

33. Lana, A. et al. An updated climatology of surface dimethlysulfide concentrations and emission fluxes in the global ocean. *Global Biogeochem. Cycles* **25**, GB1004 (2011).
